# Matching target dose to target organ

**DOI:** 10.12688/f1000research.10055.2

**Published:** 2017-03-30

**Authors:** Desmond I. Bannon, Marc A. Williams

**Affiliations:** 1 Toxicology Directorate, Health Effects Division, U.S. Army Public Health Center, Edgewood, MD, USA

**Keywords:** In vitro, lead, in vivo, dose, stem cells.

## Abstract

*In vitro* assays have become a mainstay of modern approaches to toxicology with the promise of replacing or reducing the number of *in vivo* tests required to establish benchmark doses, as well as increasing mechanistic understanding. However, matching target dose to target organ is an often overlooked aspect of *in vitro* assays, and the calibration of *in vitro* exposure against *in vivo* benchmark doses is often ignored, inadvertently or otherwise.  An example of this was recently published in *Environmental Health Perspectives* by Wagner *et al *(2016), where neural stems cells were used to model the molecular toxicity of lead.  On closer examination of the *in vitro* work, the doses used in media reflected *in vivo* lead doses that would be at the highest end of lead toxicity, perhaps even lethal.  Here we discuss the doses used and suggest more realistic doses for future work with stem cells or other neuronal cell lines.

A recent article by Wagner *et al.* reported the involvement of the anti-oxidant Nrf2 transcription factor signaling pathway in the toxicity of lead using neural stem cells in an *in vitro* model of neuronal differentiation ^1^. While this work was completed in a similar way to other studies involving *in vitro* lead exposure, the work avoids a critical, often neglected issue of what constitutes a relevant physiological dose *in vitro*. The assumption that the selected dose of 1 µM (or 20.7 µg/dL) for neuronal stem cell exposure was “4 times the CDC levels of concern (LOC) for blood lead (5 µg/dL) and is within the range of exposed populations” requires further examination. Since the *in vitro* exposure was completed in media (the equivalent of plasma or serum) and not in whole blood, the assumption that the *in vitro* lead level would be equivalent to that found in whole blood of lead-exposed humans is somewhat inaccurate. Lead in serum (or plasma) represents only a fraction (~1%) of the level found in whole blood ^2, 3^, with the major fraction of lead bound inside erythrocytes ^4^. For arguments sake, if the proportion of lead used in this study was 1% of that in whole blood, the extrapolated blood lead value would be approximately 2073 µg/dL, a level over 400 times the CDC LOC, and one that would be acutely toxic and perhaps lethal.

Another study, which was cited by Wagner *et al.* ^1^, showed that measurable effects in stem cells *in vitro* could occur at doses as low as 0.4 µM ^5^; this dose would represent a blood lead level of 829 µg/dL, using the same assumptions as above. In a study by Chan *et al.,* the lowest dose of 1 µM lead used in a study of newborn rat neuronal stem cells would represent 20.73 µg/L in serum and a systemic blood lead level of about 2073 µg/dL ^6^. Other studies examining the toxicity of lead in cell cultures have also failed to adequately match the *in vitro* doses ^7– 9^ with those found *in vivo*, by taking account of the well documented relationship between plasma and whole blood lead values. More importantly, with measurable effects only beginning at greater than 10 µM for some studies ^6, 9^, could these data suggest the alternative conclusion - that neuronal cells *in vivo* are more resistant to toxic insult by lead, at least in the short term?

What is clear is that at current blood lead levels in the US population, serum or plasma levels will represent a very low fraction of those values and *in vitro* work could more realistically model neurological effects in humans if target doses were better matched to target organ. Thus, the model of exposure proposed by Wagner *et al.* and other *in vitro* work demonstrating toxic effects of lead ^5– 9^ may be more appropriate for high acute exposures. More realistically, to ensure that doses used for *in vitro* assays are complimentary to a target *in vivo* blood lead level of 20 µg/dL, exposure to cells *in vitro* should correspond to ~1% of the cited blood lead value, or a dose of 0.2 µg/dL (0.01 µM). At the current CDC 5 µg/dL LOC for children, the *in vitro* dose would become 0.05 µg/dL (0.002 µM); a dose that would present difficulties to laboratories that cannot eliminate background levels from residual lead on glassware and other sources of possible contamination or confounding of the reported data. Background contamination in controls would mean requiring higher exposure doses to demonstrate an effect, essentially making the assays less sensitive.

In the study by Wagner *et al.* ^1^, much of this may have been considered by the authors, and key assumptions may have been made; however, the question still remains whether the upregulation of genes in the Nrf2-mediated anti-oxidative stress pathway would have been observed if a more physiologically relevant dose of 0.2 µg/dL (0.1 µM) in the media (i.e., representing a blood lead level of 20 µg/dL) had been used.

How does lead in plasma compare to lead in cerebrospinal fluid? Presumably the plasma fraction contains the lead moiety that interacts with molecular targets in the brain. Evidence shows that lead in cerebrospinal fluid is 50% of that in serum ^2^, indicating that the assumptions made here are consistent with target doses of lead in the brain being much closer in value to plasma than to whole blood lead. We did not account of the evidence that the proportion of lead in plasma increases with increasing blood lead value ^3, 4^ – which could affect our upward extrapolations from putative plasma values of 20 µg/dL to whole blood lead levels of 2073 µg/dL – but it should not affect extrapolating downward to plasma lead from a starting blood lead of 20 µg/dL as the relationship between whole blood and plasma lead seems to be linear in that region ^3^. However, even if we used a value of 5% lead in plasma the extrapolated blood lead for the Wagner *et al.* study would turn out to be 20-fold the plasma which is 400 µg/dL.

Our article raises questions about what a relevant *in vitro* lead dose should be when it is contextually related to *in vivo* blood lead values. A scan of the literature for this article has shown that there are a significant number of *in vitro* publications using lead that lack (or even misinterpret) context with whole blood lead levels, thereby identifying molecular effects that may not have relevance to current national blood lead values. We propose that matching target dose to target organ should be more carefully considered with future *in vitro* work.

## Disclaimer

The views expressed in this article are those of the author(s) and do not necessarily reflect the official policy of the Department of Defense, Department of the Army, U.S. Army Medical Department or the U.S.
