# Supplementary material for: Matching target dose to target organ
Source: F1000Res. 2017 Mar 30;5:2785. Originally published 2016 Nov 29. [Version 2] doi: 10.12688/f1000research.10055.2 (PMC5270582; doi:10.12688/f1000research.10055.2)
Supplement: Peer review history

# Peer review: Referee response for version 1

**DOI:** 10.5256/f1000research.10834.r19115

**Published:** 2017-01-26

**Authors:** Donald Smith

**Affiliations:** 1 Microbiology and Environmental Toxicology, University of California Santa Cruz, Santa Cruz, CA, USA

This commentary is well written, very well justified, and timely. While there are countless published papers on the myriad effects of lead in biological systems, the consideration of dose extrapolation from *in vitro* to *in vivo* studies and their relationships to the human condition often goes unappreciated. Indeed, since toxicology is driven by the dose of the poison, establishing environmental or occupational relevance of the dose is absolutely key to the relevance of the findings. This commentary points this out in a concise and evidence-driven fashion, and is worthy of publication.

 Below are a few minor comments to consider.

  

1. Pg. 2, 1 ^st^ para: *For arguments sake, …*
  Comment: A caveat here might be that is known that the proportion of whole blood lead in plasma increases with increasing blood lead, so it is likely that the blood lead level that would produce a 1 uM plasma lead would be lower than 2,073 ug/dL, but this does not detract from the point the authors are making, which is a good and important one.
2.  Pg. 2, 3 ^rd^ para: *Thus, the model proposed in this and other work…*  
  Comment: It is not clear whose work 'this work' is referring to - Chan *et al*?
3. Pg. 2, 3 ^rd^ para: *To ensure that doses used in in vitro assays are complimentary to a target in vivo blood lead level of 20 μg/dL…*
  Comment: This suggestion by the authors is reasonable, assuming that plasma lead reflects extracellular fluid lead, though it might also be worth looking at the relationship between blood lead and CSF lead levels (in the literature) to see if it follows an appx 1% relationship as does plasma to further substantiate this suggestion.
4. Pg. 2, 3 ^rd^ para: *…eliminate background levels from residual lead on glassware and other sources of possible contamination or confounding of the reported data…*
  Comment: This too raises an important point in that the vast majority of studies do not make sufficient effort to reduce background lead levels in control cultures, so it is quite possible that here and in those other studies the control cultures, even with modestly elevated background lead levels will also be affected, requiring higher exposure doses to demonstrate a difference or 'effect' in the lead-exposed treatments. It is good that the authors pointed this out.

I have read this submission. I believe that I have an appropriate level of expertise to confirm that it is of an acceptable scientific standard.

# Response

**Published:** 2017-03-02

**Authors:** Mark A Williams

**Affiliations:** Army Public Health Center, USA

Reviewer 2.   We thank reviewer #2 for knowledgeable and helpful comments on our article. Here are our responses to specific comments. 

 Comment 1.  This point is well made – we agree that the proportion of lead in plasma would increase as blood lead increases, so that equivalent plasma lead at blood lead values greater than 100 µg/dL could be upwards of 2%. As it was we selected 1% plasma/blood ratio as the blood lead under question was 20 µg/dL but of course there is some inbuilt error in our calculations at high doses. Nonetheless, our extrapolated exposure scenario is meant to demonstrate that the assumptions under which many *in vitro* studies lie with respect to their relationship to *in vivo* blood lead values are often violated; the reviewer also acknowledges our efforts to point this out. We have added more text to acknowledge this non-linear relationship at increasing doses between whole blood lead and plasma lead.   

 Comment 2.  This sentence has been restructured to indicate that we referring to the Wagner *et al* study, as well as other studies that have made similar assumption.  

 Comment 3.  We agree that cerebrospinal fluid measures would further corroborate our assumptions. The work by Manton *et al* (cited in our article) showed that cerebrospinal fluid levels were about 50% of serum levels, though it should be pointed out that this work was carried out in only one subject. We have added more text to acknowledge this fact. 

 Comment 4.  We agree with the further elaboration of this sentence and have added additional text to incorporate the details of the comment.

# Peer review: Referee response for version 1

**DOI:** 10.5256/f1000research.10834.r19455

**Published:** 2017-01-18

**Authors:** Mir Ahamed Hossain

**Affiliations:** 1 Johns Hopkins University School of Medicine, Department of Neurology, Baltimore, MD, USA

*In vitro* assays have become a mainstay of modern approaches to toxicology with a high promise of understanding the underlying mechanisms of toxicity. The results reported by Wagner *et al.*, (2016) in the August 26 issue of the Environmental Health Perspectives, where neural stem cells were used to model the toxicity of lead. The results support the notion that lead treatment of cells leads to upregulation of vascular gene expression (JBC 275:27874-27882, 2000). While this work presents interesting effects, this reviewer’s opinion is in agreement with the correspondence (critiqued article) authors Bannon and Williams that it may be more appropriate for high acute exposures particularly in case of neural stem/progenitor cells, which lack many of the characteristic features of mature neurons.   

 It is also likely that neural stem cells (NSCs) could be more resistance to toxic insult by lead - at least in the short term. Thus the *in* *vitro* work could more realistically model chronic neurological effects if doses are better matched with the doses at the target site, as supported by the fact that serum or plasma levels represent a very low fraction of the total blood lead levels. Thus the concentrations of lead used in this study, which elicits upregulation of genes in the Nrf2-mediated anti-oxidative stress pathway, appear to be in the low micromolar range, which is much higher than the *in* *vitro* dose equivalent of the current CDC levels of concentrations (5 ug/dL) for children. Thus the concentrations used in the study does not reflect the likely exposure of lead in the environment, that is to say, concentrations which are likely to be cytotoxic particularly in case of NSCs. This is clearly a near impossible issue to address empirically, but if some information available along these lines using a more physiologically relevant dose in the media of *in* *vitro* NSC cultures to show gene expression in the Nrf2-mediated anti-oxidative stress pathway would be helpful for the reader as suggested by Bannon and Williams in the critiqued article. It will also be interesting to see how the differentiated neurons from lead exposed NSCs express neurons specific features or exhibit mature neuronal function.

I have read this submission. I believe that I have an appropriate level of expertise to confirm that it is of an acceptable scientific standard.

# Response

**Published:** 2017-03-02

**Authors:** Mark A Williams

**Affiliations:** Army Public Health Center, USA

Reviewer 1.  We thank reviewer #1 for helpful comments on our article. We address some specific aspects below.

 The reviewer agreed with our principle argument, but goes on to state that the Wagner *et al* “results support the notion that lead treatment of cells leads to upregulation of vascular gene expression”, citing an *in vitro* microarray study using astrocytes, (Hossain *et al*, 2002, ref 9 above) when in fact two of the three VEGF transcripts listed in Supplemental Table 1 of Wagner *et al* were downregulated by lead, with only one – VEGFA downregulated by 0.8-fold – being statistically significant. Therefore the cited publication by Hossain *et al* is contradicted by the Wagner *et al* data for the VEGF gene.

 The fact that the Hossain *et al* study used 10 µM lead acetate to dose astrocytes *in vitro* further supports our main point – that most lead concentrations *in vitro* would reflect highly lethal lead concentrations *in vivo* if the difference between lead in whole blood (red blood cells) and plasma were taken into account. Hossain *et al* did cite Audersirk (Audesirk G, *et al.*  In Vitro Cell Dev Biol. 1989 Dec;25(12):1121-8) as supporting evidence for the use of 10 µM lead as a dosing solution for astrocytes, where Audersirk measured free lead (Pb ^2+^) in the nanomolar range in the presence of full experimental media dosed with micromolar lead acetate using an ion selective electrode. However, Audersirk’s work in snail and chick neurons did not examine the potential lethality of the *in vitro* working doses to the whole organism, taking account of plasma/whole blood differences.
